# COVID-19 pandemic global impact on children´s health in Cameroon

**DOI:** 10.11604/pamj.2021.39.5.24304

**Published:** 2021-05-03

**Authors:** Dominique Enyama, Diomède Noukeu Njinkui, Jeanne Mayouego Kouam, Christian Eyoum, Danièle Christiane Kedy Koum, Séraphin Nguefack

**Affiliations:** 1 Department of Pediatrics, Faculty of Medicine and Pharmaceutical Sciences, University of Dschang, Dschang, Cameroon,; 2 Department of Ophthalmology, Douala Laquintinie Hospital, Douala, Cameroon,; 3 Department of Clinical Sciences, Faculty of Medicine and Pharmaceutical Sciences, University of Douala, Douala, Cameroon,; 4 Department of Pediatrics, Faculty of Medicine and Biomedical Sciences, University of Yaoundé I, Yaoundé, Cameroon

**Keywords:** COVID-19, impact, child health, Cameroon

## To the editors of the Pan African Medical Journal

From the beginning of the COVID-19 outbreak as of April 18^th^, 2021, over 140 730 596 confirmed cases have been diagnosed in 192 countries with 3 011 014 deaths. In Cameroon, the first patient was diagnosed on March 5^th^, 2020 and as of April 18^th^, 2021, there were 64 809 confirmed cases and 939 deaths [1]. During the acute phase of the outbreak, it was commonly pointed up that, children were less susceptible to develop severe forms of the disease. However, despite this fact, the COVID-19 pandemic has numerous other issues that may negatively impact children´s health. Early estimates of the indirect effects of the COVID-19 pandemic in low-income and middle-income countries predicted an increase in maternal and infant mortality in case of a reduction in the population's access to health care [2]. In Cameroon, a survey among pediatricians showed that pediatric outpatient consultations dropped significantly from 60.4% of pediatricians seeing more than 30 patients per week before the pandemic to 9.9% during the pandemic (p < 0.000) [3]. Another study showed that mortality doubled during the months of April and May 2020 with 9.9% and 11.2% of hospital deaths respectively, compared to 4.9% (p < 0.0008) and 5.1% (p < 0.0001) during the same period of the previous year [4]. This drop in attendance has led to delays in emergency and follow-up consultations and therefore in the care of children with acute and chronic conditions resulting in increased mortality and morbidity. In this context of social distancing and declining attendance at health facilities, telehealth constitutes a possible approach to reduce this gap between sick children and caregivers. It is urgent that it should be structured and promoted to keep the caregiver-patient bond.

The drop in health facilities attendance by children is also accompanied with a decrease in the use of preventive medicine care such as vaccination with the risk of an upsurge in vaccine-preventable diseases [5]. In Cameroon, an immunization campaign against poliomyelitis coupled with the distribution of mosquito-nets, initially planned for the month of April 2020, had been postponed in some regions of the country because of the COVID-19 outbreak. In most of the countries concerned, one of the strategies to fight the pandemic was global containment and the closure of schools and universities. Several experimental and observational studies emphasize that sedentary behavior, and in particular “TV time”, are associated with higher obesity and cardio-metabolic risks in children and adolescents [6]. One of the measures in the Cameroon government strategic plan against COVID-19 that aimed to specifically address the burden of the pandemic on children was the closure of schools and universities from March 18^th^, 2020 to May 31^st^, 2020. This has imposed an unusual sedentary lifestyle, especially for school-aged children, adolescents and young adults. The impact of sedentarity on child health (obesity, cardiovascular diseases) following this period is currently unknown. On the other hand, the reopening of schools and universities in Cameroon on the 1^st^ June 2020 was framed by general measures that aimed at limiting the spread of the virus, including washing hands, wearing masks and limiting the number of children in classrooms. It is possible that compliance with these measures in this specific pediatric population has limited the transmission of infection in schools in particular, but to our knowledge, there is actually no published work on the rate of COVID-19 contamination in schools in Cameroon.

Another major issue is the impact of the outbreak on children’s mental health. Some authors have addressed the aspects concerning children´s mental health during COVID-19 pandemic [7, 8]. Many studies reported negative psychological effects including post-traumatic stress symptoms, confusion, and anger. Stressors may include long quarantine duration, infection fears, frustration, boredom, inadequate supplies, inadequate information, financial loss, and stigma [9]. Generally, the care for sick adults requires, among other measures, isolation or quarantine, during which the child, separated from his parent, will be taken care of by other adults. Sometimes the death of the sick parent can occur and the child ends up in turmoil. Another aspect to take into account would be the social representations around COVID-19 and the impact on families and children. Finally, children's mental health also largely depends on that of their parents. Sick and overwhelmed parents may not be able to cope with adversity. Child abuse identification and prevention during the lockdown is an issue that is suggested by some authors [10]. In difficult times, where children and adults have to spend the days at home, tensions can arise and lead to an increase in domestic violence and abuse. An important aspect is to identify these situations and better, to prevent them.

The impact of the COVID-19 pandemic on children´s health is not only directly linked to COVID-19, but also to the suffering generated by all of the indirect effects of the pandemic on the social and family environment of the child. Taking into account the therapeutic and acute aspects of the care of children during the pandemic should not obscure a more global approach to other issues. The organization of care should be inclusive and take into account the somatic impact as well as the psychological and socio-cultural aspects.

## References

[1] Dong E, Du H, Gardner L (2020). An interactive web-based dashboard to track COVID-19 in real time. Lancet Infect Dis.

[2] Roberton T, Carter ED, Chou VB, Stegmuller AR, Jackson BD, Tam Y (2020). Early estimates of the indirect effects of the COVID-19 pandemic on maternal and child mortality in low-income and middle-income countries: a modelling study. Lancet Glob Health.

[3] Enyama D, Chelo D, Noukeu Njinkui D, Mayouego Kouam J, Fokam Djike Puepi Y, Mekone Nkwele I (2020). Impact of the COVID-19 pandemic on pediatricians' clinical activity in Cameroon. Arch Pediatr.

[4] Chelo D, Mekone Nkwelle I, Nguefack F, Mbassi Awa HD, Enyama D, Nguefack S (2021). Decrease in Hospitalizations and Increase in Deaths during the Covid-19 Epidemic in a Pediatric Hospital, Yaounde-Cameroon and Prediction for the Coming Months. Fetal Pediatr Pathol.

[5] Bramer CA, Kimmins LM, Swanson R, Kuo J, Vranesich P, Jacques-Carroll LA (2020). Decline in Child Vaccination Coverage During the COVID-19 Pandemic-Michigan Care Improvement Registry, May 2016-May 2020. MMWR Morb Mortal Wkly Rep.

[6] Margaritis I, Houdart S, El Ouadrhiri Y, Bigard X, Vuillemin A, Duché P (2020). How to deal with COVID-19 epidemic-related lockdown physical inactivity and sedentary increase in youth? Adaptation of Anses' benchmarks. Arch Public Health.

[7] Jiao WY, Wang LN, Liu J, Fang SF, Jiao FY, Pettoello-Mantovani M (2020). Behavioral and Emotional Disorders in Children during the COVID-19 Epidemic. J Pediatr.

[8] Liu JJ, Bao Y, Huang X, Shi J, Lu L (2020). Mental health considerations for children quarantined because of COVID-19. Lancet Child Adolesc Health.

[9] Brooks SK, Webster RK, Smith LE, Woodland L, Wessely S, Greenberg N (2020). The psychological impact of quarantine and how to reduce it: rapid review of the evidence. Lancet.

[10] Ramaswamy S, Seshadri S (2020). Children on the brink: Risks for child protection, sexual abuse, and related mental health problems in the COVID-19 pandemic. Indian J Psychiatry.

